# Advances in Modelling and Simulation of Materials in Applied Sciences

**DOI:** 10.3390/ma18174141

**Published:** 2025-09-04

**Authors:** Evaggelos Kaselouris

**Affiliations:** 1 Physical Acoustics and Optoacoustics Laboratory, Department of Music Technology and Acoustics, Hellenic Mediterranean University, 74133 Rethymnon, Greece; vagfem@hmu.gr; 2 Institute of Plasma Physics and Lasers-IPPL, University Research and Innovation Centre, Hellenic Mediterranean University, 74150 Rethymnon, Greece

Advances in materials science, engineering, and computer science have created new opportunities for physicists and engineers to develop novel methods for material processing and characterization. This has led to a greater need for advanced modeling and simulation techniques that can capture multiphysics phenomena across various spatial and temporal scales. As a result, modeling and simulation have become essential tools, working alongside experimental measurements to deepen our understanding of materials.

In recent years, significant progress has been achieved in computational approaches such as finite element simulations [1,2,3,4,5,6,7,8,9,10], multiphysics simulations [11,12,13,14,15,16,17,18], ab initio and molecular dynamics simulations [19,20,21,22,23,24,25], and structural optimization algorithms [26,27,28,29,30,31,32]. Applications span a wide spectrum of engineering fields, including mechanical and manufacturing engineering [33,34,35,36,37,38,39,40,41,42,43,44,45,46], mechanical behavior and characterization of materials [47,48,49,50,51,52,53,54,55,56,57,58,59,60], thermomechanics [16,17,18,61,62,63,64,65,66] and thermohydrodynamics [67,68,69], vibration and acoustics [70,71,72,73,74,75,76,77], and fluid dynamics [78,79,80,81,82,83,84]. Materials under study range from metals and alloys to composites, metamaterials, and multifunctional structures. This enlarged bibliography highlights the breadth of modelling and simulation approaches and highlights the effective integration of computational and experimental methods, showing that simulations are not just for predicting outcomes but also for guiding the design, processing, and optimization of materials.

Within this broad context, the contributions collected in this Special Issue (Refs. [85,86,87,88,89,90,91,92,93,94]) can be grouped into five specific topics that illustrate how these general advances are applied to cutting-edge problems in materials science. These are as follows:

(i) Finite element simulations in mechanical and manufacturing engineering: Advanced roll forming of dual-phase steels [85] and optimization of corrugated tubes for crashworthiness [86].
(ii) Mechanical behavior and characterization of materials: Integration of digital image correlation for accurate property determination [87]; fibrous material remodeling under stress [88]; lightweight and multifunctional structures, including TPMS sandwich cores [89] and tunable phononic crystals [90].
(iii) Reliability and vibration performance of engineered systems: Fatigue life prediction of composite hydrogen storage vessels under random vibration [91].
(iv) Thermohydrodynamic multiphysics modelling: Simulation of plasma arc melting for titanium alloys [92].
(v) Ab initio, atomistic modelling of materials: Short-range ordering in gallium melts [93] and first principle calculations in Cu–Zn alloys [94].

Song and co-authors [85] performed finite element simulations using Abaqus to investigate the roll forming process of a wheel rim made from high-strength dual-phase steel DP590 following flash butt welding. The study modeled the flaring, three-roll forming, and expansion stages, incorporating weld- and heat-affected zone microstructural data to analyze stress–strain distribution, thickness reduction, and forming stability. Results showed stress and strain concentrations in welded and coarse- or fine-grained heat-affected zones, with significant thickness reduction in groove and flange sections due to softening in intercritical and subcritical HAZ regions. Microstructural features, such as layered martensite and coarse grains, played a decisive role in deformation behavior. Process optimization, including a recommended friction coefficient of 0.4 and an optimal feed-to-roll speed ratio of 15 mm/s to 1:1.2, improved forming quality. The findings highlight the need for careful application of DP590 in rim manufacturing and suggest broader potential in automotive, aerospace, and rail sectors, as well as opportunities for advanced simulation methods, parameter refinement, and microstructure control to enhance performance and durability.

To address the limitations of longitudinal corrugated tubes (LCTs) and circumferential corrugated tubes (CCTs) in crashworthiness, a novel bi-directional corrugated tube (BCT) was developed in the study of Zou and co-authors [86], combining the high specific energy absorption of CCTs with the stable platform force of LCTs. Formed by rolling a bi-directional corrugated structure into a circular tubular form, the BCT was evaluated through experiments and finite element simulations, with results showing strong agreement. Parametric studies and multi-objective optimization revealed that tailored geometric parameters significantly improve energy absorption and stability, with the BCT achieving up to 80% higher specific energy absorption than CCTs and 45% higher than LCTs, while reducing ultimate load capacity. These findings position the BCT as a promising energy absorber for applications in automotive crash boxes, aerospace structures, and other fields requiring enhanced crashworthiness, with future work focusing on high-speed impact and vibration behavior.

Accurate mechanical property parameters are essential for reliable finite element simulations, and the study of Wang and co-authors [87] compared strain gauge and digital image correlation (DIC) techniques in tensile testing of 316 L stainless steel used in pressurized water reactor pipelines. Results showed that while both methods yield nearly identical data within the strain gauge’s range, DIC offered a far broader measurement scope, capturing complete strain evolution and enhancing elastoplastic simulation accuracy. Finite element predictions calibrated with DIC data closely matched experimental strain distributions, validating DIC as a powerful tool for both parameter acquisition and simulation verification. Its wider applicability positions it as a promising method for characterizing complex, heterogeneous nuclear structures such as welded joints or defect-containing components, where traditional gauges face spatial limitations.

Rodella [88] presented a continuum model for fibrous materials, like those in biological tissues and composites, that undergo plastic remodeling under mechanical stress. The research identified three distinct classes of material behavior based on how their internal fibers reorient under tension. This reorientation led to complex and extreme responses, including phase segregation (where different regions of the material develop unique fiber alignments) and transitions between auxetic and non-auxetic properties. The findings, derived from a combination of analytical and numerical methods, offer significant implications for engineering new materials with tunable properties for applications ranging from soft robotics and protective equipment to biomedical implants.

Triply periodic minimal surface (TPMS) sandwich structures offer promising lightweight, high-strength, and energy-absorbing solutions, and the study of Vasile and co-authors [89] evaluated eight novel TPMS cores and a stochastic topology using finite element analysis, using Ansys, with both implicit and conventional modeling approaches. While implicit modeling proved as accurate as traditional CAD-based methods, it required up to four times greater computational effort. Sensitivity analyses identified optimal meshing parameters, and four material models for SLA-manufactured photopolymer parts were assessed, with the Multilinear model achieving the highest accuracy at only 2.54% deviation from experimental results. Numerical simulations closely matched experimental failure mechanisms for most cases, validating the predictive capability of the approach. The findings highlight implicit modeling as a viable tool for optimizing complex geometries before fabrication, offering valuable guidance for designing high-performance sandwich structures in engineering applications.

The study of Kaniolakis Kaloudis and co-authors [90] investigated how unit cell multiplicity influenced the acoustic response of phononic crystals (PCs), using a laser–plasma sound source characterization method combined with computational multiphysics simulations. Results revealed that increasing cell multiplicity deepened phononic bandgaps, exponentially attenuated acoustic energy in the first bandgap, and enhanced spectral flatness in passband regions, while also increasing the number of resonant peaks tied to spherical harmonics and standing wave modes. A methodology was proposed to determine the number of unit cells required to achieve a specific bandgap depth, especially with respect to the behavior of a structure with infinite length. These findings underscore the tunability of PCs for targeted sound control and noise insulation applications, and set the stage for future studies on defected cells, higher-order structures, and material density effects.

Xiong and co-authors [91] presented an experimental–numerical investigation of composite hydrogen storage vessels (CHSVs) under random vibration, a key reliability concern for fuel cell vehicle applications. Finite element models, validated against modal testing with <9% frequency deviation, were used to predict stress distribution and fatigue life via Steinberg’s and Dirlik’s methods. Results identified the head section as the critical fatigue zone, with initial maximum stresses of 469.4 MPa (winding layer) and 173.0 MPa (liner), corresponding to lifetimes of ~1.66 × 10^6^ and 3.06 × 10^6^ cycles. Optimization of ply parameters reduced stresses by 66% and 85%, respectively, and extended both components’ lifetimes to over 1 × 10^7^ cycles, meeting high-cycle fatigue standards. The findings provide design and optimization guidelines applicable to a broad class of CFRP–metal or CFRP–polymer pressure vessels.

The study by Bellot and co-authors [92] combined advanced numerical modeling and pilot-scale experiments to evaluate inclusion removal in titanium alloys processed via Plasma Arc Melting–Cold Hearth Remelting (PAMCHR), a key route for producing aeronautical-grade materials. Using a custom PAM3D model on the Ansys-Fluent platform, the work simulated thermohydrodynamic behavior, melting rates, and high-density inclusion (HDI) trajectories in a 100–150 kg/h pilot furnace. Multiphysics simulations, validated by crucible bath profile and residence time measurements, revealed that over 50% of torch-supplied heat is lost through crucible cooling and that bath depth reached approximately one-third of the metal height in the crucible. Both modeling and HDI insemination tests confirmed efficient sedimentation-based removal of refractory inclusions—an advantage over processes like Vacuum Arc Remelting. These results provide a predictive tool for process optimization and highlight PAMCHR’s potential for reliably producing ultra-clean titanium alloys for critical aerospace applications.

Wang and co-authors [93] employed the Wulff cluster model (WCM) to elucidate the short-range ordering (SRO) structure of gallium melts, revealing clusters with Wulff-shaped exteriors and crystallographic symmetry interiors. High-temperature XRD measurements at 523 K, 623 K, and 723 K showed excellent agreement with Ab initio simulations in peak positions, widths, and relative intensities, even well above the melting point. Minor deviations at 523 K are linked to pre-nucleation onset. By integrating experimental data, DFT-derived surface energies, and structural modelling, the study confirms WCM’s capability to describe SRO in melts with partial covalent character, such as gallium, providing insight into melt structure–property relationships.

The study of Huang and co-authors [94] combined first-principles calculations with the Boltzmann transport equation to investigate electrical and thermal conductivities of Cu–Zn alloys across α, α + β′, and β′ phases with 0–50 at.% Zn. Results revealed a non-monotonic decrease–increase trend in both properties, consistent with experiments. Electronic structure analysis showed that Cu d-orbitals dominated near the Fermi level in the α-phase, with increasing Zn lowering the effective density of states and reducing electron transport. Thermal conductivity trends, validated via the Wiedemann–Franz law, aligned with measured data. These findings provide theoretical insight into transport mechanisms and optimization strategies for complex alloy systems.
